# Foliar Application of Bamboo-Derived Nano-Biochar Enhances Morphological and Biochemical Responses of Lettuce (*Lactuca sativa* L.) Under Salt Stress

**DOI:** 10.3390/plants15010009

**Published:** 2025-12-19

**Authors:** Bhornchai Harakotr, Sompop Taebuanhuad, Yaowapha Jirakiattikul, Thanpisit Puangchick

**Affiliations:** Department of Agricultural Technology, Faculty of Science and Technology, Thammasat University, Pathum Thani 12120, Thailand; sompop.tae@dome.tu.ac.th (S.T.); yjirakia@tu.ac.th (Y.J.); pthanpis@tu.ac.th (T.P.)

**Keywords:** salinity tolerance, antioxidant defense system, ion balance, osmotic adjustment, carbon-based foliar amendments, sustainable agriculture

## Abstract

Nano-biochar (n-BC) is an emerging eco-friendly material with potential to improve crop performance under salt stress. This study aimed to evaluate the effects of foliar applications of bamboo-derived n-BC on the morphological and biochemical responses of lettuce plants under salt stress (40 mM NaCl). n-BC solutions (1.0, 3.0, and 5.0% *w*/*v*) were foliar-applied every five days until harvest. Salt stress markedly increased hydrogen peroxide (H_2_O_2_) and malondialdehyde (MDA) by 264.54% and 14.02%, disrupted Na^+^/K^+^ homeostasis, and reduced biomass. Foliar n-BC mitigated these effects by reducing Na^+^ accumulation by 22.24–25.11% and enhancing K^+^, Ca^2+^, and Mg^2+^ uptake. The treatments also improved photosynthetic pigments and increased proline, soluble proteins, and soluble sugars. Oxidative damage was alleviated, as reflected by reductions in H_2_O_2_ and MDA together with enhanced ascorbate peroxidase, catalase, and superoxide dismutase activities. Total phenolics, flavonoids, and ABTS and DPPH scavenging activities also increased under n-BC application. Among all the concentrations, 3.0% (*w*/*v*) n-BC consistently produced the greatest improvements in growth, ionic balance, and antioxidant responses. These findings demonstrate that bamboo-derived n-BC is a promising foliar biostimulant for enhancing lettuce performance under saline conditions.

## 1. Introduction

Environmental change has become a major challenge for crop production in meeting the increasing global demand for food. Moreover, a substantial proportion of arable land is now subjected to various stress conditions [1]. Abiotic stresses such as salinity, heat, drought, flooding, and heavy metal toxicity hinder plant growth and significantly reduce crop yields [2]. Especially, salt stress adversely affects all stages of plant development and ultimately reduces crop yield [3,4]. This is largely due to reduced water availability and osmotic stress, which induce physiological drought and impair essential cellular functions [5]. Excessive Na^+^ and Cl^−^ accumulation further disrupts ion homeostasis and metabolic activity, leading to impaired photosynthesis, stomatal closure, chlorosis, and membrane damage [6,7]. Moreover, salinity-driven ionic imbalance intensifies the production of reactive oxygen species (ROS), such as superoxide radical (•O_2_^−^) and hydrogen peroxide (H_2_O_2_), which induces oxidative stress and cellular injury [6,8]. Numerous strategies have been proposed to mitigate salinity stress and enhance crop productivity, including improving ionic homeostasis, strengthening antioxidant defense systems, and supporting photosynthetic performance, all of which are essential for resilient crop production under saline conditions [3]. Consequently, research efforts are increasingly focusing on environmentally friendly approaches that can effectively support these physiological processes.

The rapidly advancing field of nanotechnology has made considerable strides in agriculture, offering innovative and timely solutions vital for sustainable farming. Nanoparticles (NPs) have emerged as a promising approach to enhancing plant nutrition [8]. This progress in agricultural nanotechnology is driven by its potential to deliver targeted solutions that improve crop growth, yield, and stress tolerance. Studies indicate that nanoparticles can mitigate the adverse effects of abiotic stresses, such as salinity, by enhancing nutrient uptake and promoting plant adaptations [8,9,10]. Nano-biochar (n-BC) is an emerging nanostructured, carbon-rich, and highly aromatic material synthesized from biomass sources such as agricultural residues, municipal waste, and animal manure with high carbon content [9,10]. n-BC is produced by first converting biomass into biochar (BC) through pyrolysis at 300–700 °C under limited oxygen, followed by modification methods such as chemical treatment, ball milling, or gaseous activation. The resulting n-BC particles, typically 10–100 nm in size, are black and nanoscale in structure. Compared to conventional BC, n-BC exhibits enhanced physicochemical properties, including a greater surface area, higher microporosity, stronger ion-exchange capacity, and improved adsorption efficiency [8,10,11]. Furthermore, the morphology and physicochemical characteristics of n-BC can be tailored through material selection, modification techniques, catalysts, solvents, and processing temperatures to optimize its foliar uptake efficiency and targeted physiological functions [9,12]. Bamboo is an ideal crop for n-BC production due to its rapid growth, low nutrient requirements, and strong root system that helps prevent erosion and improve soil water retention. Compared to woody plants, bamboo provides a more efficient and environmentally friendly biomass source for regenerative agriculture and renewable energy. Additionally, bamboo thrives without the need for intensive cultivation, making it a low-maintenance and sustainable option [13,14,15].

In agricultural applications, both BC and n-BC are commonly used as soil amendments due to their long half-life, which can range from hundreds to thousands of years, thereby reducing carbon release from soil to the environment [16]. Their incorporation enhances soil fertility and decreases the mobility of toxic heavy metals [17,18]. Additionally, some studies have reported improved crop productivity and mitigation of both soil-borne and airborne plant diseases following their application [13,19]. Consequently, they have emerged as promising tools in the effort to use alternative biofertilizers for increasing crop production and maintaining environmental security. Several studies have shown that achieving effective results with these materials often requires high application rates, typically between 10 and 50 t ha^−1^, depending on the soil and biochar properties [18]. Foliar applications of BC and n-BC have emerged as a promising alternative, offering beneficial effects on plant growth and physiological traits while potentially reducing the quantity of material required compared to conventional soil amendments [17,18,20,21]. However, these nanoscale features provide specific advantages when applied as a foliar fertilizer, such as improved leaf surface adhesion, faster penetration through stomata or cuticular pathways, and more efficient delivery of ions and bioactive compounds. For instance, Khaliq et al. [17] reported that combining soil and foliar applications of n-BC significantly enhanced the storage root biomass of carrot compared to soil application alone. Moreover, the foliar application of n-BC has been demonstrated to produce the most pronounced enhancements in growth and yield parameters across various crops under stress conditions [8,20,22,23]. n-BC enhances plant stress tolerance by strengthening membrane stability, improving water status, and activating antioxidant defenses [8,22,24,25]. Studies show that nano-biochar can upregulate superoxide dismutase (SOD), peroxidase (POD), and catalase (CAT) activities, leading to reduced ROS accumulation, lower lipid peroxidation, and decreased electrolyte leakage [8,22]. n-BC has been linked to osmolyte regulation and nutrient-related processes in several crops, with direct evidence for its effects on proline, ascorbic acid, and Na^+^/K^+^ homeostasis [26]. However, crop responses vary depending on the type of n-BC, its synthesis method, and the application rate [8]. Moreover, limited research has evaluated the effects of foliar-applied bamboo-derived n-BC in alleviating salt stress in leafy vegetables via enhanced metabolite accumulation and antioxidant enzyme activities.

Lettuce (*Lactuca sativa* L.) is categorized into horticultural types—iceberg, romaine, butterhead, and leaf varieties—that meet diverse consumer demands, ranging from whole heads to baby leaf mixes [27]. This crop is considered relatively sensitive to salinity, exhibiting reduced germination, disrupted membrane integrity, redox imbalance, and impaired photosynthesis under salt stress [2,28]. For this reason, lettuce is commonly used as a model species for investigating the effects of salinity stress in plants. We hypothesized that foliar-applied bamboo-derived n-BC would enhance lettuce’s tolerance to salinity by improving ionic balance, boosting antioxidant defenses, and strengthening photosynthetic and metabolic functions, resulting in improved overall plant growth. Therefore, the objective of this research was to evaluate the effects of foliar-applied bamboo-derived n-BC on the morphological and biochemical responses of lettuce under salt stress. Our findings provide clear physiological and biochemical evidence of the mechanisms through which n-BC enhances salinity tolerance, underscoring its potential to improve leafy vegetable production.

## 2. Results

### 2.1. Characteristics and Physicochemical Properties of BC and n-BC

**Figure 1:**
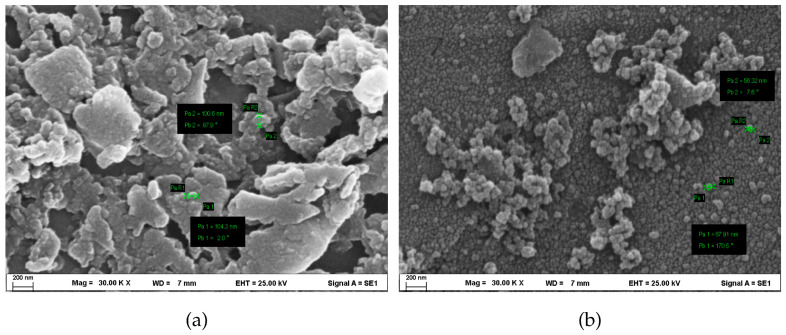
Scanning electron microscope micrograph of BC (**a**) and n-BC (**b**) (30 k× magnification).

Scanning electron microscopy (SEM) confirmed that ball milling efficiently reduced the BC (Figure 1a) into uniformly dispersed nanosized particles (Figure 1b), with diameters ranging from 56.3 to 67.9 nm. This result is consistent with the nanoscale classification and was further supported by dynamic light scattering (DLS) analysis, which showed a comparable hydrodynamic particle size distribution (Figure S1). Simultaneously, the SEM morphology and DLS measurements validate that the processed material used in this study can be reliably regarded as n-BC. The n-BC displayed a globular morphology with higher circularity indices (Figure 1b), in contrast to the angular and sharp-edged structure observed in BC (Figure 1a). An energy-dispersive X-ray spectroscopy (EDS) analysis indicated the presence of characteristic signals for carbon, oxygen, and nitrogen in both BC and n-BC, confirming these as the primary elemental compositions (Table S1). All the analyzed metals were detected in both samples, except for phosphorus, which was absent from the BC. All the analyzed heavy metals—Hg, Cd, Cr, and Ni—were below their respective detection limits in both BC and n-BC. The pH of BC was 8.59, reflecting strong alkalinity, whereas conversion to n-BC lowered the pH to 6.81 (Δ pH = −1.785). Similarly, the electrical conductivity (EC) significantly decreased from 0.610 to 0.595 dS m^−1^, indicating reduced ionic leaching potential following nanoscale transformation.

**Figure 2:**
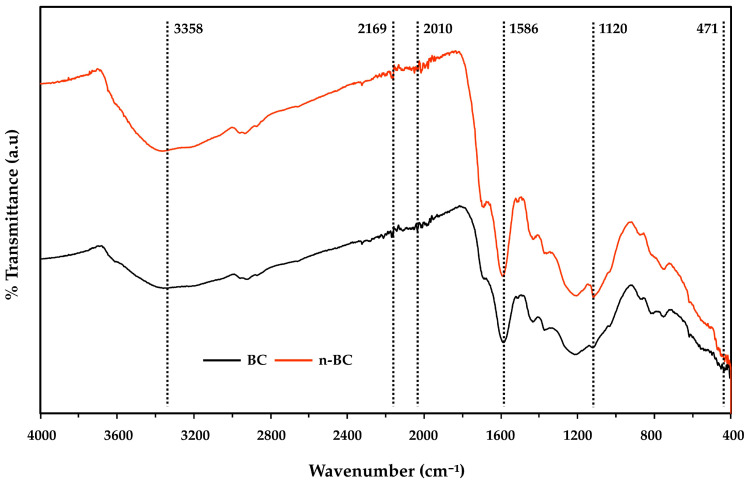
FTIR spectra of BC (black line) and n-BC (red line).

The FTIR spectra of BC and n-BC were highly similar (spectral similarity index R^2^ > 0.95), with characteristic absorption bands observed in both materials (Figure 2). The spectra exhibited a broad absorption band around 3421 cm^−1^, attributed to O–H stretching vibrations (strongly intensity). A medium absorption at 1697 cm^−1^ was assigned to the C=O stretching of carboxyl (–COOH) groups, while bands in the 1160–1030 cm^−1^ range corresponded to C–O–C stretching vibrations of polysaccharide derivatives. Additional peaks included aromatic ring vibrations at ~870 cm^−1^ and a low–frequency metal-oxide stretching band at ~422 cm^−1^. Notably, in n-BC, a weak band near 2160 cm^−1^ appeared, tentatively assigned to unsaturated groups (C≡C or C≡N), which was absent in BC. Furthermore, a medium-intensity band at ~471 cm^−1^ was attributed to Si–O/Si–C stretching, consistent with silica phytolith residues in bamboo feedstock.

### 2.2. Morphological Attributes

**Figure 3:**
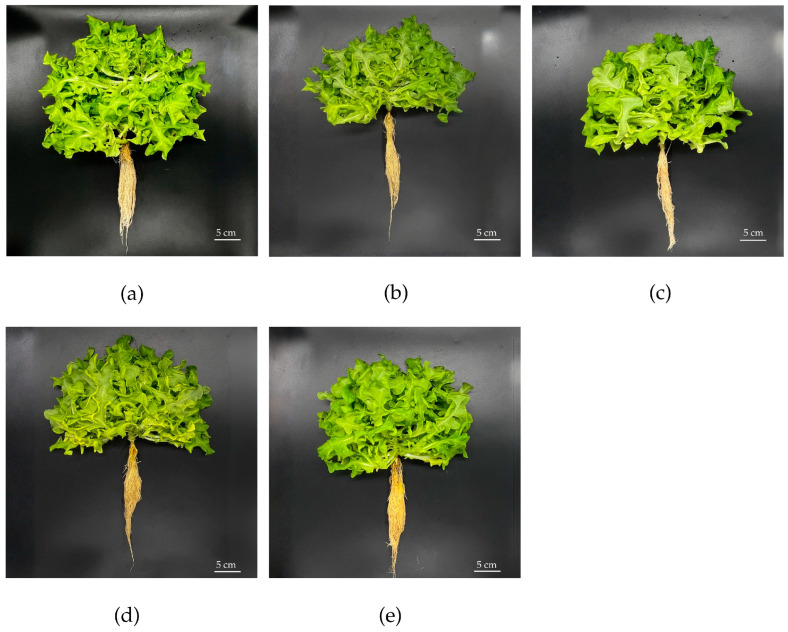
The appearance of Green Oak lettuce grown under salt stress conditions at 35 DAT, as influenced by the foliar application of n-BC. (**a**) Nutrient solution, leaf sprayed with distilled water; (**b**) nutrient solution containing 40 mM NaCl, leaf sprayed with distilled water; (**c**–**e**) nutrient solution containing 40 mM NaCl, leaf sprayed with of 1.0, 3.0, and 5.0% (*w*/*v*) n-BC, respectively.

**Figure 4:**
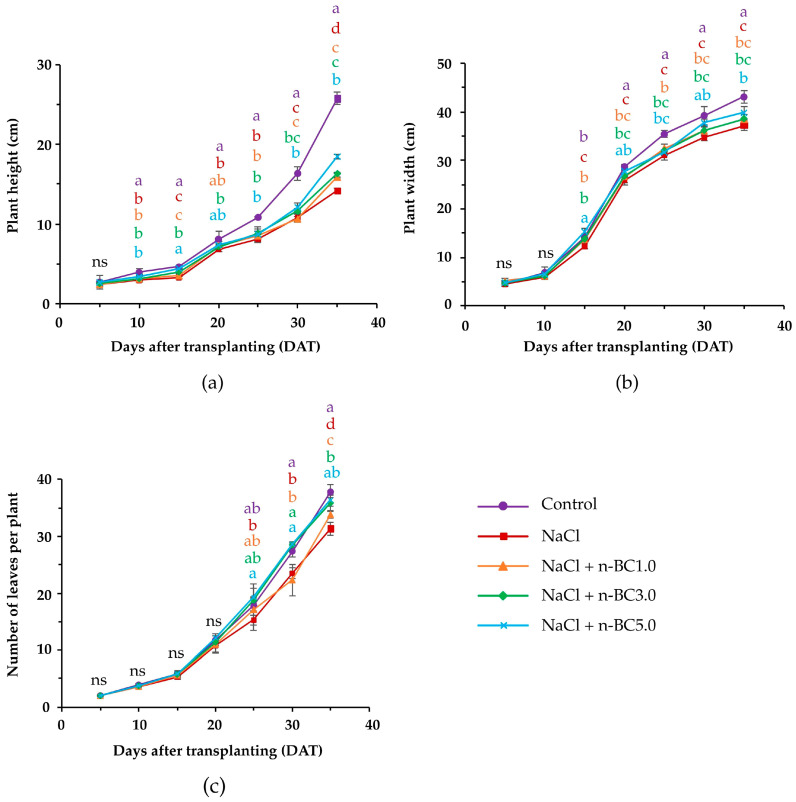
Growth performance of lettuce grown under salt stress between 5 and 35 DAT, as influenced by the foliar application of n-BC. (**a**) Plant height; (**b**) plant width; (**c**) number of leaves per plant. Control: nutrient solution, leaf sprayed with distilled water; NaCl: nutrient solution containing 40 mM NaCl, leaf sprayed with distilled water; NaCl + n-BC1.0, NaCl + n-BC3.0, and NaCl + n-BC5.0; nutrient solution containing 40 mM NaCl, leaf sprayed with of 1.0, 3.0, and 5.0% (*w*/*v*) n-BC, respectively. No significant differences were observed among treatments sharing the same lowercase letters on the same day after transplantation, according to the LSD test at the 5% significance level.

Figure 3 illustrates the appearance of Green Oak lettuce grown under salt stress following the foliar application of n-BC. Salt stress significantly inhibited the growth of lettuce, as evidenced by notable reductions in the plant height, plant width, and number of leaves per plant compared to the control group (Figure 4). However, foliar applications of 3.0 or 5.0% (*w*/*v*) n-BC significantly improved these growth parameters at 35 days after transplanting (DAT). The fresh and dry weights of lettuce under NaCl treatment alone were lower compared with those in the control groups (Table 1). The application of n-BC significantly enhanced lettuce shoot and root biomass compared to the NaCl treatment alone, but not the shoot fresh weight, which was not significantly affected by 5.0% (*w*/*v*) n-BC. All treatments with n-BC increased the dry weight of the shoots and roots compared to the control. The most pronounced effect was observed for the 3.0% (*w*/*v*) concentration, with increases ranging from 117% to 139%.

### 2.3. Photosynthetic Pigment Contents

The results showed that NaCl treatment significantly reduced the contents of chlorophyll b (Chl b), total chlorophyll (TChl), and carotenoids (Caro) by 0.77, 0.18, and 5.57%, respectively, compared with the control, whereas the change in chlorophyll a (Chl a) was not significant (Table 2). In contrast, foliar applications of 3.0 and 5.0% (*w*/*v*) n-BC increased the Chlb content compared with NaCl treatment alone and exhibited no significant difference from the control group. Moreover, these n-BC treatments enhanced Caro content by 1.47 and 3.38%, respectively, relative to the NaCl treatment. The 3.0% (*w*/*v*) n-BC application produced the greatest improvement in both Chla and TChl contents, exceeding those of both the control and NaCl-treated plants (by 0.53–0.58% and 0.36–0.54%, respectively). Although the mean differences in pigment traits were small, they exceeded the LSD threshold at the 5% level, and the very low standard deviations reflect the high consistency of the controlled extraction and spectrophotometric procedures.

### 2.4. Cation Contents

The results showed that NaCl treatment increased the Na^+^ content while reducing K^+^ accumulation (by 492.31 and 62.40%, respectively), resulting in a 37.62% higher Na^+^/K^+^ ratio compared to the control (Table 3). However, the application of 3.0 and 5.0% (*w*/*v*) n-BC reduced Na^+^ levels and the Na^+^/K^+^ ratio (by 22.24–25.11% and 56.44–65.34%, respectively), and enhanced K^+^ accumulation (by 69.43 and 118.34%, respectively). NaCl treatment significantly decreased Ca^2+^ and Mg^2+^ accumulation in lettuce compared to the control (by 79.41 and 25.00%, respectively). The application of 3.0% (*w*/*v*) n-BC led to the greatest enhancement in both ion contents (by 253.51 and 25.93%, respectively). The increase in Mg^2+^ content at this n-BC level was not significantly different from that of the control.

### 2.5. Osmolytes, H_2_O_2_, and Malondialdehyde (MDA) Contents

NaCl treatment enhanced proline accumulation and soluble sugar levels in lettuce plants but had no significant effect on soluble protein content (Table 4). Among all the treatments, the foliar application of 3.0% (*w*/*v*) n-BC led to the highest increases in proline, soluble sugar, and soluble protein levels compared to plants subjected to NaCl stress alone (by 40.67, 115.60, and 187.94%, respectively). On the other hand, NaCl treatment significantly increased free radical accumulation and oxidative damage, as indicated by elevated levels of H_2_O_2_ and MDA compared to the control (by 264.54 and 14.02%, respectively). However, n-BC application markedly reduced these oxidative stress markers, with 3.0% (*w*/*v*) n-BC leading to the greatest reduction in H_2_O_2_ and MDA accumulation (75.38 and 30.70%, respectively).

### 2.6. Antioxidant Defense Systems

Under salt stress, plant cells upregulate a complex antioxidant defense system, including enzymes such as ascorbate peroxidase (APX), CAT, and SOD, which act to neutralize ROS. It was observed that NaCl treatment slightly improved the activities of these enzymatic antioxidants in lettuce compared to the control by 101.39, 84.94, and 19.20%, respectively (Table 5). Increasing trends in the activities of APX, CAT, and SOD were observed with the application of high levels of n-BC, whereas 1.0% (*w*/*v*) n-BC1.0 did not result in a significant difference in these enzyme activities compared to the NaCl treatment. Especially, 3.0% (*w*/*v*) n-BC led to the greatest enhancement in the activities of these enzymes (by 70.82, 41.32, and 23.23% above the NaCl group), except that CAT activity was not significantly different from that under 5.0% (*w*/*v*) n-BC (by 43.16% above the NaCl group).

Salt stress significantly enhanced the accumulation of non-enzymatic antioxidants in lettuce, as indicated by increases in total phenolic content (TPC), total flavonoid content (TFC), and ABTS antioxidant activity compared to the control group of 23.10, 12.48, and 46.37%, respectively (Table 6). However, no significant difference was observed in antioxidant activity according to the DPPH assay. The application of n-BC positively influenced these parameters compared to both the control and NaCl-only treatments. Treatment with 3.0% (*w*/*v*) n-BC resulted in the greatest overall improvement (by 83.63, 30.88, 37.82, and 55.83% above the NaCl group). However, the TFC and antioxidant activity according to the ABTS assay were not significantly affected by the 5.0% (*w*/*v*) n-BC (approximately 30% above the NaCl group).

Salt stress also appeared to induce the synthesis of representative phenolic acids and flavonoids in lettuce compared to the control, except for chlorogenic acid (CGA), ferulic acid (FA), and quercetin (QE), which did not show significant differences (Table 7). The application of most n-BC treatments also enhanced the accumulation of these antioxidants compared with both the control and NaCl-only treatments, with 3.0% (*w*/*v*) n-BC leading to the highest increases in CGA, caffeic acid (CFA), and *p*-coumaric acid (*p*-CA) of 24.83, 24.56, and 20.25%, respectively. In contrast, ferulic acid (FA), cinnamic acid (CA), and rutin (RT) did not differ significantly among the n-BC treatments. Additionally, QE was not detected at 3.0 and 5.0% (*w*/*v*) n-BC, indicating a concentration-dependent metabolic response.

### 2.7. Principal Component Analysis (PCA)

**Figure 5:**
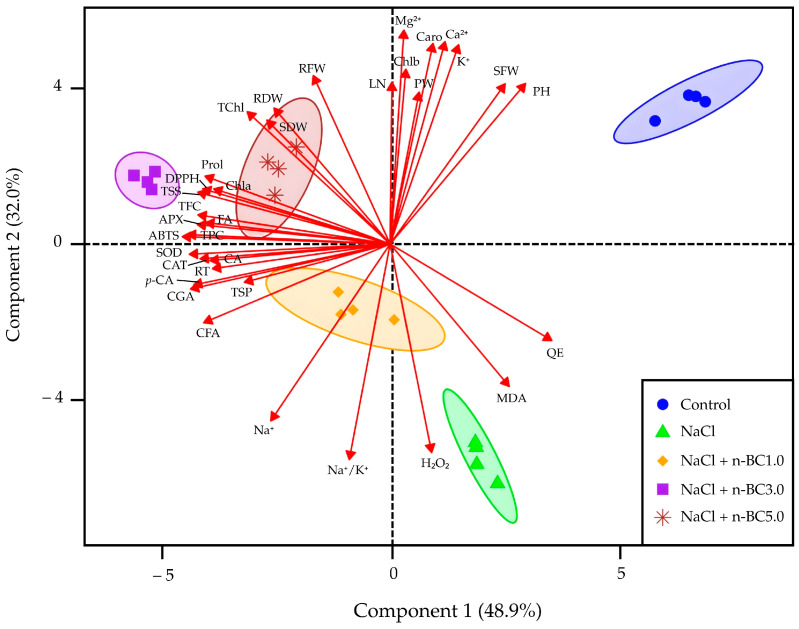
PCA score and loading plots of morphological and biochemical attributes of lettuce grown under salt stress and treated with n-BC. Treatments: Control: nutrient solution, leaf sprayed with distilled water; NaCl: nutrient solution containing 40 mM NaCl, leaf sprayed with distilled water; NaCl + n-BC1.0, NaCl + n-BC3.0, and NaCl + n-BC5.0: nutrient solution containing 40 mM NaCl, leaf sprayed with of 1.0, 3.0, and 5.0% (*w*/*v*) n-BC, respectively. PH: plant height; PW: plant width; NL: number of leaves per plant; SFW: shoot fresh weight; SDW: shoot dry weight; RFW: root fresh weight; RDW: root dry weight; Chla: chlorophyll a; Chlb: chlorophyll b; TChl: total chlorophyll; Caro: carotenoid; Prol: proline; TSS: total soluble sugar; TSP: total soluble protein; H_2_O_2_: hydrogen peroxide; MDA: malondialdehyde; APX: ascorbate peroxidase; CAT: catalase; SOD: superoxide dismutase; TPC: total phenolic content; TFC: total flavonoid content; ABTS: ABTS antioxidant activity; DPPH: DPPH antioxidant activity; CGA: chlorogenic acid; CFA: caffeic acid; FA: ferulic acid; *p*-CA: *p*-coumaric acid; CA: cinnamic acid; RT: rutin; QE: quercetin.

A PCA biplot with a 95% confidence ellipse was constructed for 35 dependent variables to examine the relationships among morphological and biochemical attributes in lettuce grown under salt stress and treated with n-BC. The PCA score and loading plots revealed clear treatment-dependent separation, with PC1 (48.9%) and PC2 (32.0%) explaining 80.9% of the total variation (Figure 5). NaCl-stressed plants clustered distinctly with high positive loadings of H_2_O_2_ and MDA, along with negative loadings of Na^+^ and the Na^+^/K^+^ ratio, indicating strong contributions of oxidative and ionic stress variables to PC1. In contrast, the control group grouped with traits such as plant height, plant width, shoot fresh weight, leaf number, Caro, Chlb, and nutrient ions (K^+^, Ca^2+^, and Mg^2+^), which loaded positively on PC1 and PC2, reflecting healthy physiological status. The foliar application of n-BC shifted plants away from stress-associated vectors, with the 3% (*w*/*v*) n-BC treatment forming the tightest cluster aligned with high negative loadings of antioxidant enzymes (APX, CAT, and SOD), phenolics, flavonoids, soluble sugars, and ABTS and DPPH activities. The 5% (*w*/*v*) n-BC treatment was associated with Chla, TChl, proline, and biomass traits, whereas 1% (*w*/*v*) n-BC showed intermediate positioning. Overall, the combined score-loading structure confirms that n-BC improved lettuce physiological and biochemical responses in a dose-dependent manner, with 3% (*w*/*v*) n-BC exerting the strongest influence on antioxidant and metabolic recovery under salinity.

According to Pearson’s correlation coefficients (Table 8), QE (r = 0.77) showed strong positive correlations with PC1, whereas ABTS (r = −0.97), APX (r = −0.96), SOD (r = 0.95), *p*-CA (r = −0.95), CGA (r = −0.93), TPC (r = −0.92), TFC (r = −0.92), total soluble sugar (r = −0.91), DPPH (r = −0.89), proline (r = −0.88), CFA (r = −0.88), FA (r = −0.88), CA (r = −0.85), Chla (r = −0.84), and RT (r = −0.84) exhibited highly negative correlations. These variables contributed substantially to the variance explained by the first principal component. For PC2, Mg^2+^ (r = 0.96), Ca^2+^ (r = 0.91), Car (r = 0.90), K^+^ (r = 0.89), root fresh weight (r = 0.76), number of leaves per plant (r = 0.73), shoot fresh weight (r = 0.72), and plant height (r = 0.72) displayed strong positive correlations. In contrast, the Na^+^/K^+^ ratio (r = −0.97), H_2_O_2_ (r = −0.94), and Na^+^ (r = −0.79) exhibited strong negative correlations. These variables represent a distinct and independent dimension of variability captured by the second principal component, likely associated with morphological and biochemical attributes not encompassed by PC1.

## 3. Discussion

n-BC is a novel nanostructured material derived from BC, which is produced through biomass pyrolysis and subsequently processed using various top-down techniques and physical degradation methods [21]. Ball milling is a widely used technique for reducing BC into nanoparticles or NPs (<100 nm), resulting in increased surface area, microporosity, hydrophobicity, and functional group availability [12]. In this study, BC was produced from bamboo, a high-biomass-yield species with a rapid growth rate [29], and subsequently converted into n-BC using ball milling for 36 h. SEM–EDS was employed to examine the individual particles and confirm the size of n-BC, indicating that the morphology of n-BC particles became more rounded and compact, with an average particle size between 56.3 and 67.9 nm (Figure 1). Similar morphological characteristics of n-BC have been reported in studies utilizing high-speed and planetary ball milling of oil palm biomass [12] and feedstocks [30], respectively. Elemental analysis revealed that the surface of n-BC was primarily composed of C, O, and N (Table S1), according to other studies [30]. Interestingly, the C, O, and N contents of n-BC were lower than those of the original BC, possibly due to the greater difficulty in breaking chemical bonds within the BC structure [30]. Moreover, no heavy metals were detected in either BC or n-BC (Table S1), although certain BC derived from other feedstocks may contain elevated Cd, Ni, or Cr [31]. After ball milling, the pH of n-BC shifted from alkaline (8.59) to near neutral (6.80), likely due to the loss or exposure of alkaline functional groups [32].

FTIR analysis indicated that adsorption in both BC and n-BC primarily involves surface complexation among functional groups, with O–H, C–O–C, and aromatic ring vibrations as the dominant features (Figure 2). Minor differences were observed, such as a weak peak in the n-BC spectrum suggesting the presence of alkynes (triple bonds) and a medium-intensity band at 471 cm^−1^ attributed to Si–O and Si–C stretching vibrations. These differences highlight that, while the overall chemical framework of BC remains intact, ball milling can introduce subtle structural modifications. Variability in FTIR outcomes across studies [12,30,33] may be due to differences in biomass feedstocks and pyrolysis conditions.

In the present study, visible symptoms of salt stress in lettuce plants included reduced growth performance and decreased shoot and root fresh and dry weights (Figure 3 and Table 1), which are consistent with previous studies [28,34,35,36]. However, our results demonstrated that the foliar application of bamboo-derived n-BC significantly improved the growth performance of lettuce, a salt-sensitive crop, under salt stress conditions. This may be because the high surface reactivity and negative zeta potential of n-BC enhance adhesion to the leaf surface, stomatal entry, and subsequent symplastic and apoplastic translocation, thereby improving the distribution of absorbed particles [17,37,38]. This phenomenon is attributed to the elevated N, P, and K concentrations in both shoot and root tissues, suggesting that foliar-applied n-BC facilitated nutrient uptake and redistribution [28]. As the primary component of n-BC, nano-carbon enhances nutrient absorption by facilitating water and mineral uptake, thereby improving plant growth and biomass accumulation, as reported in tomato [8] and rice [39]. The greatest improvements were observed with a 3.0% (*w*/*v*) n-BC application, identifying this concentration as optimal for lettuce growth (Table 1), consistent with the findings of Rasheed et al. [22], who reported maximal carrot growth at the same concentration under stress conditions, while 1.0% (*w*/*v*) was suboptimal and 5.0% (*w*/*v*) excessive. Moreover, we found that 5.0% (*w*/*v*) n-BC also improved growth relative to the NaCl treatment, though the growth did not surpass that at 3.0% (*w*/*v*), suggesting a threshold beyond which foliar-applied nanoparticles may hinder rather than enhance nutrient fluxes. Excessive NPs may induce oxidative stress, generate ROS, and hinder nutrient uptake due to particle aggregation [40]. This highlights the importance of crop species; developmental stage; and the type, particle size, or dosage of n-BC in influencing plant responses to its application.

Chlorophyll and carotenoids are essential for light harvesting and photoprotection, and their reduction under salt stress is commonly attributed to ROS-mediated degradation [41,42]. In the present study, NaCl treatment significantly decreased the contents of chlorophylls and carotenoids compared with the control, except for Chla, which was not significantly affected (Table 2). Salt stress also suppressed the accumulation of photosynthetic pigments and reduced photosynthetic parameters, including the net photosynthetic rate, stomatal conductance, transpiration rate, and water use efficiency, ultimately leading to photoinhibition [36]. However, the foliar application of n-BC markedly increased chlorophyll and carotenoid contents in lettuce under salt stress. Especially, the 3.0% (*w*/*v*) n-BC treatment exhibited the greatest mitigation of pigment loss. This finding is consistent with previous reports in tomato, where n-BC alleviated salt-induced pigment degradation [8]. The enhanced pigment stability observed here is likely due to n-BC-induced activation of antioxidant enzymes and mitigation of oxidative stress, which preserve chloroplast integrity and sustain pigment biosynthesis under salt stress [8,43]. Overall, these results suggest that n-BC application plays a pivotal role in mitigating the salt-induced inhibition of photosynthetic pigment synthesis.

NaCl treatment disturbed ionic balance in lettuce plant by increasing Na^+^ accumulation and reducing K^+^ uptake, which led to a higher Na^+^/K^+^ ratio (Table 3). The foliar application of n-BC counteracted these effects by promoting K^+^ uptake and limiting Na^+^ accumulation, in agreement with reports in spinach and rice [22,44]. Foliar-applied NPs can enter leaves through stomata or the cuticle and move via vascular tissues to the roots, where they influence nutrient uptake and root growth [45,46]. They also initiate systemic signaling through hormones and messengers such as ROS and calcium waves, which can stimulate root development, limit Na^+^ transport to shoots, and promote vacuolar Na^+^ sequestration [45,46,47]. The presence of Si in n-BC (0.18%, Table S1) might also contribute by reducing Na^+^ mobility and facilitating K^+^ uptake. In addition, n-BC increased Ca^2+^ and Mg^2+^ levels compared with salt-stressed plants, which could help stabilize membranes and contribute enzyme activity. Moreover, ball milling increased the Ca and Mg contents of n-BC compared with BC (Table S1), and foliar n-BC accordingly elevated these ions in lettuce. While the specific mechanisms require further clarification, n-BC appears to support ionic homeostasis under salinity by enhancing nutrient delivery and regulating Na^+^/K^+^ balance. Its near-neutral pH, resulting from surface oxidation during ball milling, likely improves ion exchange and foliar nutrient absorption, further contributing to ionic balance restoration.

Under NaCl treatment, lettuce accumulated more osmolytes, with proline showing the most distinguished increase (Table 4). Proline is well known as a major osmoprotectant that contributes to redox balance, photosynthesis, and stress recovery [36,48]. The foliar application of n-BC further elevated proline, soluble proteins, and soluble sugars compared with NaCl alone, with the strongest effect at 3.0% (*w*/*v*). A comparable trend was observed in tomato, where foliar n-BC increased amino acids and soluble sugars under both stress and non-stress conditions [8]. These changes are likely associated with improved nitrogen metabolism and carbohydrate turnover, processes previously associated with nitrate reductase activity and photosynthetic efficiency [49,50]. Although these mechanisms were not directly assessed here, they provide a reasonable explanation for the observed increase in osmolyte levels. Finally, our findings indicate that foliar-applied n-BC promotes osmolyte accumulation, which helps maintain osmotic adjustment and alleviates the impact of salinity in lettuce.

Salt stress caused oxidative damage in lettuce, as reflected by higher H_2_O_2_ and MDA contents (Table 4), which agrees with earlier reports [22,36,51]. The foliar application of n-BC lowered both parameters, similar to the reductions observed in spinach and tomato [5,19]. The presence of Si in n-BC (Table S1) may have contributed to this response because silicon nanoparticles are known to enhance antioxidant activity [52]. Under NaCl treatment, the APX and CAT activities increased while SOD remained unchanged (Table 5). When 3.0% (*w*/*v*) n-BC was applied, all three enzymes increased significantly, suggesting stronger enzymatic detoxification, consistent with previous observations [8,22,53]. NPs are known to stimulate antioxidant defenses such as APX, SOD, and CAT under abiotic stress [44]. Consistent with the enzymatic antioxidant results, the foliar application of n-BC in our study also reduced ROS accumulation (H_2_O_2_ and MDA) by 264.54% and 14.02%, respectively, compared with the NaCl group. Nevertheless, direct measurements of Si uptake and related gene expression are required to clarify the underlying mechanisms. Non-enzymatic antioxidants (TPC, TFC) and radical-scavenging activities (ABTS, DPPH) also increased in response to n-BC, with the highest values again at 3.0% (*w*/*v*) (Table 6). Similar patterns have been described in tomato, where foliar n-BC elevated flavonoid content [8]. Overall, these results suggest that n-BC enhances both enzymatic and non-enzymatic antioxidant systems, helping lettuce handle oxidative stress under salinity.

Phenolic acids and flavonoids are important contributors to ROS detoxification and plant defense against abiotic stress [22,54]. In our study, NaCl stress increased the levels of several phenolics in lettuce, such as CFA, *p*-CA, CA, and RT, which agrees with reports from other crops [54,55]. The foliar application of n-BC further stimulated the accumulation of these compounds, except for QE, and the strongest effect was observed at 3.0% (*w*/*v*) (Table 7). Especially, CGA, which is the predominant phenolic in lettuce [28,56,57], was significantly elevated by n-BC treatment, reinforcing its role in ROS detoxification and membrane protection [58]. These changes likely reflect activation of the phenylpropanoid pathway, in which phenylalanine ammonia-lyase (PAL) acts as a central enzyme [54]. The presence of Si in n-BC (Table S1) may also play a role, since Si has been reported to enhance PAL activity in basil treated with Si nanoparticles [59,60]. Moreover, QE was not detected in the 3.0 and 5.0% (*w*/*v*) n-BC treatments, likely because it was adsorbed onto the high-surface-area n-BC via π–π stacking, hydrogen bonding, or hydrophobic interactions, thereby reducing its measurable free form [61]. This finding suggests that n-BC can affect flavonoid stability and bioavailability. Therefore, our results indicated that foliar n-BC enhances phenolic metabolism, thereby improving the antioxidant capacity of lettuce under salt stress.

The PCA clearly separated the treatments and showed that n-BC distinctly modulates key morphological and biochemical responses under salinity (Figure 5). Lettuce exposed to NaCl was clearly separated by high positive contributions of H_2_O_2_ and MDA and by negative contributions of Na^+^ and the Na^+^/K^+^ ratio. These loadings show that PC1 is driven mainly by oxidative and ionic stress responses, consistent with well-documented mechanisms of salinity-induced ROS production, membrane lipid peroxidation, and ion disequilibrium in leafy vegetables [2,3,4]. By contrast, control plants grouped with chlorophyll pigments, Caro, biomass, and nutrient ions (K^+^, Ca^2+^, and Mg^2+^), reflecting intact photosynthetic function and stable nutrient acquisition under non-saline conditions [2,8,62]. Foliar-applied n-BC shifted the NaCl-stressed groups away from stress-associated loadings, indicating effective mitigation. The 3.0% (*w*/*v*) n-BC treatment formed the most compact cluster aligned with high loadings of antioxidant enzymes (APX, CAT, and SOD), phenolics, flavonoids, soluble sugars, and ABTS and DPPH activities, suggesting coordinated enhancement of both enzymatic and non-enzymatic antioxidant defenses. These responses agree with reports that nano-carbon materials enhance redox regulation, nutrient availability, and ROS-scavenging pathways [8,17,22]. The association with osmolytes (proline, soluble sugars, soluble proteins) further indicates improved osmotic adjustment and membrane stabilization under stress [8]. The 5.0% (*w*/*v*) n-BC group showed increased chlorophylls, proline, and biomass but with greater within-group variability, consistent with the dose-dependent effects of nanomaterials reported elsewhere [8]. The 1.0% n-BC treatment showed partial improvement, placing it between NaCl and higher n-BC treatments.

Pearson’s correlation analysis clarified the contribution of individual traits to the PCA structure. Strong positive correlations of QE (r = 0.77) with PC1 indicate that this axis reflects flavonoid-associated metabolic adjustment (Table 8). In contrast, the strong negative correlations of antioxidant activities (ABTS, r = −0.97; DPPH, r = −0.89), enzymatic antioxidants (APX, r = −0.96; SOD, r = −0.95), phenolic compounds (*p*-CA, CGA, TPC, TFC; r = −0.92 to −0.95), osmolytes (soluble sugars, r = −0.91; proline, r = −0.88), and Chla (r = −0.84) show that PC1 primarily represents the oxidative and metabolic responses associated with salinity and their mitigation by n-BC. These variables are consistent with known mechanisms through which plants alleviate ROS accumulation and maintain redox balance under salt stress [8,22,44]. PC2 captured a distinct dimension associated with mineral homeostasis and vegetative growth, as shown by strong positive correlations with Mg^2+^ (r = 0.96), Ca^2+^ (r = 0.91), Caro (r = 0.90), K^+^ (r = 0.89), and biomass traits. Conversely, the Na^+^/K^+^ ratio (r = −0.97), H_2_O_2_ (r = −0.94), and Na^+^ (r = −0.79) showed strong negative correlations, confirming PC2 as an indicator of ionic imbalance and oxidative stress severity [2,4,8]. The alignment of nutrient ions and growth traits with n-BC treatments highlights the role of nano-biochar in restoring ion homeostasis and supporting chloroplast stability under salinity [8,22].

The PCA and correlation matrices demonstrate that 3.0% (*w*/*v*) n-BC most effectively modulates the interconnected processes underlying salt tolerance, enhancing antioxidant capacity, promoting phenolic metabolism, improving mineral balance, and sustaining growth. These integrated effects align with previous findings on nano-engineered biochar as a foliar biostimulant that improves physiological resilience in salt-affected crops [4,8,16,17,22]. These physiological benefits highlight the relevance of n-BC for sustainable crop production, particularly in salt-affected areas where resource-efficient and environmentally friendly approaches are needed.

## 4. Materials and Methods

### 4.1. Preparation and Charactrization of BC and n-BC

Raw culm of 3-year-old bamboo (*Dendrocalamus sericeus* cl. Chang Mon) was obtained from a farmer’s field in Sa Kaeo province, Thailand. The bamboo was sliced to 2–4 cm thickness using a slicer and dried at 70 °C until reaching a constant weight; the dried plant samples were used to produce BC. The dried samples were pyrolyzed at 400 °C for 4 h in a semi-closed system with air vents using a muffle furnace (CWF 1100, Carbolite Gero, Baden–Württemberg, Germany), allowing controlled release of volatiles to produce BC. Consequently, the black mass was kept for cooling at room temperature. The sample was milled to BC powder using a variable-speed rotor mill (Pulverisette 14, Fritsch, Idar-Oberstein, Germany).

Nano-biochar (n-BC) was synthesized following the procedure described by Ng et al. [12] using a planetary ball mill (PM 100, Retsch, Haan, Germany) operated at ambient temperature. Zirconia (ZrO_2_) balls with a diameter of 2.0 mm were used as the grinding medium. Each milling vial contained 400 g of grinding medium and 20 g of BC powder, resulting in a ball-to-powder mass ratio (BPR) of 20:1. The milling process was conducted at a fixed speed of 500 rpm for a total duration of 36 h. Milling was carried out in 60 min intervals, with a 60 min rest period between each interval to prevent overheating. Finally, the n-BC was stored in airtight containers prior to analysis and use in experimental procedures.

The characterization and elemental composition analysis of the BC and n-BC were performed by SEM–EDS (Leo 1450 VP, Leo, North Billerica, MA, USA). Their FTIR spectra were observed using an FTIR Spectrometer (Nicolet^TM^ iS50, Thermo Scientific^TM^, Waltham, MA, USA) in the wavelength range of 4000–400 cm^−1^. Moreover, the particle size distribution of n-BC was determined using dynamic light scattering (DLS) (Master 3000, Malvern Panalytical, Malvern, Worcestershire, UK) to confirm nanoscale range. The pH was measured using a pH meter (Lab 855, SI Analytics, Mainz, Germany) in a 1:10 (sample-to-water) suspension, while EC was determined with an EC meter (CON 2700, Eutech Instruments Pte. Ltd., Singapore) using a 1:5 (sample-to-water) suspension.

### 4.2. Plant Materials and Experimental Design

Water suspensions of n-BC were prepared following Rasheed et al. [22] and sonicated for 10 min. Each formulation was supplemented with 1.0% (*w*/*v*) dispersing agent and 0.3% (*w*/*v*) preservative (Kawa International Chemie Co., Ltd., Bangkok, Thailand). Preliminary phytotoxicity testing, conducted as described in our previous study [56], indicated that neither the n-BC suspensions nor the dispersing and preservative agents caused chlorosis or affected plant growth, confirming that all formulations were safe for subsequent evaluation.

The experiment was conducted in a controlled-environment room at the Department of Agricultural Technology, Faculty of Science and Technology, Thammasat University, Pathum Thani, Thailand. The growth conditions were set at 25 ± 2 °C, 60 ± 2% relative humidity, 700 ppm CO_2_, and a 16 h light/8 h dark photoperiod, using LED lighting at 220 ± 1 µmol m^−2^ s^−1^ (115415.81.0–LM–20S1B, Grows Laboratory, Bangkok, Thailand) [63]. The temperature and humidity were continuously monitored with a HOBO data logger (Onset Data Logging Solutions, Bourne, MA, USA).

Green Oak lettuce (*L. sativa* L. var. Fusion) seedlings were cultivated on sponge sheets placed in plastic trays floating on a half-strength Resh Tropical Dry Summer nutrient solution under controlled conditions. When the seedlings developed four true leaves (approximately 14 days after sowing), uniform plants were selected and transplanted into 22 L hydroponic containers (46.5 × 18.0 × 31.0 cm^3^) filled with the same nutrient solution. Four plants were placed per container, and the solution was refreshed every two weeks. The main experiment was arranged based on a completely randomized design (CRD) with four replications and included the following treatment: Control (nutrient solution, leaf sprayed with distilled water); NaCl (nutrient solution containing 40 mM NaCl, leaf sprayed with distilled water); NaCl + n-BC1.0, NaCl + n-BC3.0, and NaCl + n-BC5.0 (nutrient solution containing 40 mM NaCl, leaf sprayed with 1.0, 3.0, and 5.0% (*w*/*v*) n-BC, respectively). The 40 mM NaCl concentration was selected based on our preliminary experiment, which identified this level as inducing moderate but non-lethal salinity stress in lettuce, and is consistent with the salinity range reported by Rouphael et al. [62]. The plants were allowed to grow for 6 days after transplanting (DAT) before the application of the experimental trials to ensure proper adaptation to the containers. Foliar sprays (25–50 mL container^−1^) were applied at five-day intervals until 30 DAT, using a handheld sprayer.

### 4.3. Measurements of Morphological Attributes

The plant height, plant width, and leaf number were recorded at 5 DAT and subsequently at 5-day intervals until 35 DAT. At the final harvest, two plants from each treatment were collected and separated into shoots and roots. The fresh weight was measured immediately, after which samples were oven-dried at 60 °C to constant weight for dry biomass determination. Shoots from another two plants per treatment were frozen in liquid nitrogen to halt enzymatic activity, freeze-dried, ground using a variable-speed rotor mill, sieved through a 60-mesh screen, homogenized, and stored at −20 °C until biochemical analysis.

### 4.4. Determination of Photosynthetic Pigment Content

Photosynthetic pigments were quantified following Lichtenthaler and Wellburn [64] with slight modifications. A 0.1 g mass of sample was extracted in 5 mL of 95% (*v*/*v*) ethanol and kept in darkness for 12 h. The extract was centrifuged at 15,000× *g* for 10 min at 4 °C, and the absorbance of the supernatant was measured at 665, 649, and 470 nm using a spectrophotometer (UV–1280, Shimadzu, Kyoto, Japan). The Chla, Chlb, TChl, and Caro contents were calculated and are expressed as micrograms per gram of dry weight (µg g^−1^ DW).

### 4.5. Determination of Na^+^, K^+^, Ca^2+^, and Mg^2+^ Contents

A 0.1 g mass of finely ground sample was digested in a mixed acid solution (HNO_3_:HClO_4_, 6:1, *v*/*v*) and diluted with distilled water to a final volume of 50 mL. The Na^+^, K^+^, Ca^2+^, and Mg^2+^ concentrations were determined using an atomic absorption spectrophotometer at the Soil Chemistry and Fertility Laboratory, Department of Soil Science, Kasetsart University, Thailand. The Na^+^/K^+^ ratio was subsequently calculated.

### 4.6. Determination of Proline, Soluble Sugars, and Soluble Protein Contents

The proline content was determined following Bates et al. [65]. The extract was reacted with ninhydrin and glacial acetic acid (1:1:1, *v*/*v*/*v*), incubated at 90 °C for 1 h, cooled, extracted with 2 mL of toluene, and measured at 520 nm. Soluble sugars were quantified using the phenol–sulfuric acid method of Dubois et al. [66] by reacting the homogenate with 5% phenol and concentrated H_2_SO_4_ and reading the absorbance at 485 nm. The soluble protein was determined using the Bradford assay [67]. All osmolytes are expressed as micrograms per gram of dry weight (µg g^−1^ DW).

### 4.7. Determination of H_2_O_2_ and MDA Contents

The H_2_O_2_ content was quantified according to Billah et al. [68] with slight modification. Powdered tissue (0.5 g) was homogenized in 5 mL of 0.1% (*w*/*v*) TCA and centrifuged at 10,000× *g* for 15 min. The supernatant was mixed with 2 mL of 1 M KI and 1 mL of 10 mM phosphate buffer (pH 7.0), incubated in the dark for 1 h, and measured at 390 nm.

MDA content was determined using the TBA assay [69]. A 200 mg sample was homogenized in 2 mL of 0.1% TCA and centrifuged at 12,000× *g* for 15 min, and the supernatant was mixed with 1.5 mL of 20% TCA containing 0.5% TBA. After incubation in a boiling water bath for 30 min and rapid cooling, the mixture was centrifuged at 11,180× *g* for 10 min. The absorbance was recorded at 532 and 600 nm, and the MDA concentration was calculated using an extinction coefficient of 155 mM^−1^ cm^−1^. The results are expressed as micromoles per gram of dry weight (µmol g^−1^ DW).

### 4.8. Determination of Enzymatic Antioxidants

For the APX [70] and CAT [71] assays, 0.2 g of ground tissue was homogenized in 0.5 mL of 50 mM sodium phosphate buffer (pH 7.0) containing 0.4 µg of PVP under chilled conditions. The homogenate was centrifuged at 15,000× *g* for 15 min at 4 °C, and the supernatant was used for enzyme activity determination. For SOD extraction, 0.5 g of sample was homogenized in 0.5 mL of 100 mM sodium phosphate buffer (pH 7.8) containing 0.4 µg of PVP and centrifuged using the same protocol [72]. All the antioxidant enzyme activities are expressed as units per milligram of protein (unit mg^−1^ protein).

### 4.9. Determination of Non-Enzymatic Antioxidants and Their Activities

Dried samples were extracted according to Jirakiattikul [73] using 95% ethanol at a 1:3 (*w*/*v*) ratio, with maceration repeated three times over three days. Combined extracts were filtered and concentrated to dryness using a rotary evaporator (R–300, Buchi Rotavapor^®^, Flawil, Switzerland) to obtain crude extract pellets.

The TPC and TFC were quantified using the Folin–Ciocalteu assay [74] and the method of Kubola et al. [75], respectively. The absorbance at 765 and 510 nm was recorded using a microplate reader (Power Wave XS, Biotek, San Diego, CA, USA). The results are expressed as milligrams of gallic acid equivalents per gram of dry extract (mg GAE g^−1^ DE) for TPC and milligrams of quercetin equivalents per gram of dry extract (mg QEq g^−1^ DE) for TFC.

The antioxidant activities were evaluated using ABTS [76] and DPPH [77] radical scavenging assays with absorbances at 734 and 520 nm, respectively. The results are reported as milligrams of Trolox equivalent per gram of dry extract (mg TE g^−1^ DE).

The phenolic acid and flavonoid profiles were analyzed by reversed-phase HPLC following the method of Harakotr et al. [56]. Chromatographic separation was performed using a Shimadzu HPLC system (Shimadzu Co., Ltd., Tokyo, Japan) equipped with a binary LC–20AC pump and an SPD–M20A diode array detector, and an InertSustain^®^ C18 column (250 mm × 4.6 mm, 5 μm; GL Sciences Inc., Tokyo, Japan). The mobile phase consisted of acetonitrile (solvent A) and orthophosphoric acid (solvent B, pH 2), with a flow rate of 0.5 mL/min, column temperature set at 5 °C, and injection volume of 20 μL. The detection wavelengths were 280 nm for hydroxybenzoic acids, 320 nm for hydroxycinnamic acids, and 370 nm for flavonoids. Compounds were identified by comparison with authentic standards based on retention time and UV spectra, and concentrations are expressed as milligrams per 100 g of dry extract (mg 100 g^−1^ DE).

### 4.10. Statistical Analysis

Statistical analyses were conducted using Statistix software (version 10.0; Analytical Software, Tallahassee, FL, USA). All measurements were performed using four biological replicates per treatment, and statistical tests were carried out accordingly. Analysis of variance (ANOVA) was used to evaluate treatment effects, and mean comparisons were conducted using the least significant difference (LSD) test. PCA with 95% confidence ellipses, along with Pearson’s correlation analysis between the principal components and the 35 measured variables, was performed using R software (version 4.5.1 R Core Team, Vienna, Austria).

## 5. Conclusions

Salt stress markedly restricts lettuce growth by disrupting ionic homeostasis and intensifying oxidative stress. The foliar application of n-BC, particularly at 3.0% (*w*/*v*), effectively mitigated these effects and promoted growth recovery. The protective effects of n-BC are mediated by several interconnected mechanisms. It reduced Na^+^ accumulation while restoring the balance of K^+^, Ca^2+^, and Mg^2+^. In addition, n-BC enhanced osmolyte production, including proline, soluble sugars, and proteins, and strengthened antioxidant defenses through both enzymatic (APX, CAT, and SOD) and non-enzymatic (phenolics and flavonoids, except quercetin) responses. These synchronized responses helped preserve cellular balance and ultimately supported improved growth performance under salinity stress. Although the present study was conducted under controlled conditions, the promising effects observed here suggest opportunities for further evaluation in field environments and across diverse crop species to assess scalability and practical applicability, as well as long-term safety and crop specificity.

## Figures and Tables

**Table 1:** The effect of foliar-applied n-BC on the fresh weight and dry weight of lettuce grown under salt stress at 35 DAT.

Treatments ^1^	Shoot				Root			
	Fresh Weight (g)	%	Dry Weight (g)	%	Fresh Weight (g)	%	Dry Weight (g)	%
Control	87.84 ± 5.82 ^a 2^	100	3.29 ± 0.08 ^b^	100	7.27 ± 0.53 ^bc^	100	0.33 ± 0.01 ^c^	100
NaCl	60.50 ± 4.32 ^d^	69	2.78 ± 0.13 ^c^	84	5.67 ± 0.36 ^d^	78	0.26 ± 0.02 ^d^	78
NaCl + n-BC1.0	71.14 ± 4.36 ^bc^	81	3.79 ± 0.10 ^a^	115	6.76 ± 0.45 ^c^	93	0.37 ± 0.03 ^b^	112
NaCl + n-BC3.0	73.90 ± 3.39 ^b^	84	3.85 ± 0.22 ^a^	117	8.77 ± 0.48 ^a^	120	0.46 ± 0.02 ^a^	139
NaCl + n-BC5.0	65.65 ± 3.69 ^cd^	75	3.69 ± 0.14 ^a^	112	7.51 ± 0.49 ^b^	103	0.38 ± 0.01 ^b^	115
C.V. (%)	6.13		4.13		6.43		5.08	

^1^ Control: nutrient solution, leaf sprayed with distilled water; NaCl: nutrient solution containing 40 mM NaCl, leaf sprayed with distilled water; NaCl + n-BC1.0, NaCl + n-BC3.0, and NaCl + n-BC5.0; nutrient solution containing 40 mM NaCl, leaf sprayed with of 1.0, 3.0, and 5.0% (*w*/*v*) n-BC, respectively. ^2^ Means ± standard deviations followed by the same letters in the same column are not significantly different at the 0.05 probability level as determined by LSD.

**Table 2:** The effect of foliar-applied n-BC on chlorophyll and carotenoid contents (µg g^−1^ DW) of lettuce grown under salt stress at 35 DAT.

Treatments ^1^	Chlorophyll a	Chlorophyll b	Total Chlorophyll	Carotenoid
Control	39.62 ± 0.07 ^c 2^	15.72 ± 0.02 ^a^	55.34 ± 0.06 ^c^	3.59 ± 0.03 ^a^
NaCl	39.64 ± 0.05 ^c^	15.60 ± 0.02 ^c^	55.24 ± 0.05 ^d^	3.39 ± 0.03 ^d^
NaCl + n-BC1.0	39.69 ± 0.01 ^bc^	15.66 ± 0.03 ^bc^	55.35 ± 0.03 ^c^	3.44 ± 0.02 ^c^
NaCl + n-BC3.0	39.85 ± 0.03 ^a^	15.69 ± 0.06 ^ab^	55.54 ± 0.06 ^a^	3.52 ± 0.02 ^b^
NaCl + n-BC5.0	39.75 ± 0.03 ^b^	15.68 ± 0.03 ^ab^	55.42 ± 0.02 ^b^	3.50 ± 0.01 ^b^
C.V. (%)	0.44	0.36	0.45	4.04

^1^ Control: nutrient solution, leaf sprayed with distilled water; NaCl: nutrient solution containing 40 mM NaCl, leaf sprayed with distilled water; NaCl + n-BC1.0, NaCl + n-BC3.0, and NaCl + n-BC5.0; nutrient solution containing 40 mM NaCl, leaf sprayed with of 1.0, 3.0, and 5.0% (*w*/*v*) n-BC, respectively. ^2^ Means ± standard deviations followed by the same letters in the same column are not significantly different at the 0.05 probability level as determined by LSD.

**Table 3:** The effect of foliar-applied n-BC on cation contents (% dry weight) of lettuce shoot grown under salt stress at 35 DAT.

Treatments ^1^	Na^+^	K^+^	Na^+^/K^+^	Ca^2+^	Mg^2+^
Control	0.39 ± 0.04 ^d 2^	6.09 ± 0.71 ^a^	0.63 ± 0.01 ^e^	1.36 ± 0.05 ^a^	0.36 ± 0.01 ^a^
NaCl	2.31 ± 0.12 ^a^	2.29 ± 0.11 ^d^	1.01 ± 0.06 ^a^	0.28 ± 0.04 ^e^	0.27 ± 0.02 ^d^
NaCl + n-BC1.0	2.00 ± 0.06 ^b^	2.64 ± 0.12 ^d^	0.76 ± 0.06 ^b^	0.57 ± 0.02 ^d^	0.30 ± 0.01 ^c^
NaCl + n-BC3.0	1.75 ± 0.04 ^c^	3.88 ± 0.24 ^c^	0.44 ± 0.02 ^c^	0.99 ± 0.06 ^b^	0.34 ± 0.01 ^ab^
NaCl + n-BC5.0	1.73 ± 0.09 ^c^	5.00 ± 0.01 ^b^	0.35 ± 0.02 ^d^	0.74 ± 0.11 ^c^	0.33 ± 0.02 ^b^
C.V. (%)	4.62	8.61	7.30	8.04	3.89

^1^ Control: nutrient solution, leaf sprayed with distilled water; NaCl: nutrient solution containing 40 mM NaCl, leaf sprayed with distilled water; NaCl + n-BC1.0, NaCl + n-BC3.0, and NaCl + n-BC5.0; nutrient solution containing 40 mM NaCl, leaf sprayed with of 1.0, 3.0, and 5.0% (*w*/*v*) n-BC, respectively. ^2^ Means ± standard deviations followed by the same letters in the same column are not significantly different at the 0.05 probability level as determined by LSD.

**Table 4:** The effect of foliar-applied n-BC on osmolytes, H_2_O_2_, and MDA contents of lettuce shoots grown under salt stress at 35 DAT.

Treatments ^1^	Osmolyte Contents (mg g^−1^ DW)			H_2_O_2_ (µmol g^−1^ DW)	MDA (µmol g^−1^ DW)
	Proline	Total Soluble Sugar	Total Soluble Protein		
Control	2.92 ± 0.07 ^d 2^	2.23 ± 0.02 ^e^	8.23 ± 0.39 ^c^	6.91 ± 0.59 ^d^	8.77 ± 0.61 ^b^
NaCl	3.86 ± 0.06 ^c^	2.50 ± 0.01 ^d^	10.70 ± 1.98 ^c^	25.19 ± 1.50 ^a^	10.00 ± 0.61 ^a^
NaCl + n-BC1.0	4.77 ± 0.05 ^b^	3.81 ± 0.01 ^b^	16.56 ± 2.76 ^b^	13.48 ± 2.56 ^b^	9.59 ± 0.28 ^ab^
NaCl + n-BC3.0	5.43 ± 0.18 ^a^	5.39 ± 0.02 ^a^	30.81 ± 1.96 ^a^	6.20 ± 0.52 ^d^	6.93 ± 0.67 ^c^
NaCl + n-BC5.0	4.43 ± 0.04 ^b^	3.65 ± 0.01 ^b^	17.39 ± 1.42 ^b^	10.45 ± 0.59 ^c^	6.95 ± 0.38 ^c^
C.V. (%)	2.23	0.42	11.18	11.22	7.34

^1^ Control: nutrient solution, leaf sprayed with distilled water; NaCl: nutrient solution containing 40 mM NaCl, leaf sprayed with distilled water; NaCl + n-BC1.0, NaCl + n-BC3.0, and NaCl + n-BC5.0; nutrient solution containing 40 mM NaCl, leaf sprayed with of 1.0, 3.0, and 5.0% (*w*/*v*) n-BC, respectively. ^2^ Means ± standard deviations followed by the same letters in the same column are not significantly different at the 0.05 probability level as determined by LSD.

**Table 5:** The effect of foliar-applied n-BC on ascorbate peroxidase (APX), catalase (CAT), and superoxide dismutase (SOD) (unit: mg^−1^ protein) in lettuce shoots grown under salt stress at 35 DAT.

Treatments ^1^	APX	CAT	SOD
Control	6.45 ± 0.21 ^d 2^	3.52 ± 0.47 ^c^	31.67 ± 2.53 ^d^
NaCl	12.99 ± 0.17 ^c^	6.51 ± 0.78 ^b^	37.75 ± 0.90 ^cd^
NaCl + n-BC1.0	13.39 ± 0.81 ^c^	7.36 ± 1.46 ^b^	40.59 ± 1.68 ^bc^
NaCl + n-BC3.0	22.19 ± 1.83 ^a^	9.20 ± 0.76 ^a^	46.52 ± 1.52 ^a^
NaCl + n-BC5.0	19.61 ± 0.11 ^b^	9.32 ± 0.68 ^a^	42.26 ± 1.18 ^b^
CV. (%)	6.05	21.16	4.81

^1^ Control: nutrient solution, leaf sprayed with distilled water; NaCl: nutrient solution containing 40 mM NaCl, leaf sprayed with distilled water; NaCl + n-BC1.0, NaCl + n-BC3.0, and NaCl + n-BC5.0; nutrient solution containing 40 mM NaCl, leaf sprayed with of 1.0, 3.0, and 5.0% (*w*/*v*) n-BC, respectively. ^2^ Means ± standard deviations followed by the same letters in the same column are not significantly different at the 0.05 probability level as determined by LSD.

**Table 6:** The effect of foliar-applied n-BC on the total phenolic content (TPC), total flavonoid content (TFC), and antioxidant activities in lettuce shoots grown under salt stress at 35 DAT.

Treatments ^1^	TPC	TFC	Antioxidant Activities (mg TE g^−1^ DE)	
	(mg GAE g^−1^ DE)	(mg QEq g^−1^ DE)	ABTS	DPPH
Control	5.41 ± 0.61 ^d 2^	45.66 ± 2.51 ^d^	8.13 ± 0.90 ^d^	16.68 ± 2.33 ^d^
NaCl	6.66 ± 0.47 ^c^	51.36 ± 1.29 ^c^	11.90 ± 0.35 ^c^	18.88 ± 1.53 ^d^
NaCl + n-BC1.0	10.03 ± 0.50 ^b^	61.83 ± 3.32 ^b^	14.18 ± 0.78 ^b^	21.24 ± 0.72 ^c^
NaCl + n-BC3.0	12.23 ± 0.83 ^a^	67.22 ± 3.38 ^a^	16.40 ± 0.86 ^a^	29.42 ± 0.51 ^a^
NaCl + n-BC5.0	9.09 ± 0.75 ^b^	66.98 ± 1.71 ^a^	15.52 ± 0.28 ^a^	26.38 ± 1.32 ^b^
C.V. (%)	8.62	4.91	6.01	7.35

^1^ Control: nutrient solution, leaf sprayed with distilled water; NaCl: nutrient solution containing 40 mM NaCl, leaf sprayed with distilled water; NaCl + n-BC1.0, NaCl + n-BC3.0, and NaCl + n-BC5.0; nutrient solution containing 40 mM NaCl, leaf sprayed with of 1.0, 3.0, and 5.0% (*w*/*v*) n-BC, respectively. ^2^ Means ± standard deviations followed by the same letters in the same column are not significantly different at the 0.05 probability level as determined by LSD.

**Table 7:** The effect of foliar-applied n-BC on phenolic acids and flavonoids (mg 100 g^−1^ DE) of lettuce shoots grown under salt stress at 35 DAT.

Treatments ^1^	CGA ^2^	CFA	FA	*p*-CA	CA	RT	QE
Control	7.32 ± 2.10 ^c 3^	0.99 ± 0.14 ^c^	1.14 ± 0.30 ^b^	0.61 ± 0.01 ^d^	0.33 ± 0.03 ^c^	1.17 ± 0.10 ^c^	1.03 ± 0.11
NaCl	8.78 ± 0.64 ^bc^	2.28 ± 0.17 ^b^	1.50 ± 0.33 ^b^	0.79 ± 0.05 ^c^	0.46 ± 0.05 ^b^	1.48 ± 0.15 ^b^	1.01 ± 0.08
NaCl + n-BC1.0	9.07 ± 0.61 ^b^	2.45 ± 0.40 ^b^	1.90 ± 0.19 ^a^	0.84 ± 0.04 ^bc^	0.49 ± 0.04 ^ab^	1.71 ± 0.20 ^a^	1.08 ± 0.04
NaCl + n-BC3.0	10.96 ± 0.77 ^a^	2.84 ± 0.25 ^a^	2.26 ± 0.20 ^a^	0.95 ± 0.01 ^a^	0.58 ± 0.09 ^a^	1.80 ± 0.20 ^a^	n.d. ^4^
NaCl + n-BC5.0	9.13 ± 0.43 ^b^	2.42 ± 0.23 ^b^	2.20 ± 0.14 ^a^	0.85 ± 0.03 ^b^	0.56 ± 0.05 ^a^	1.73 ± 0.08 ^a^	n.d.
C.V. (%)	12.02	11.56	13.38	4.09	10.13	9.69	4.09

^1^ Control: nutrient solution, leaf sprayed with distilled water; NaCl: nutrient solution containing 40 mM NaCl, leaf sprayed with distilled water; NaCl + n-BC1.0, NaCl + n-BC3.0, and NaCl + n-BC5.0; nutrient solution containing 40 mM NaCl, leaf sprayed with of 1.0, 3.0, and 5.0% (*w*/*v*) n-BC, respectively. ^2^ CGA: chlorogenic acid; CFA: caffeic acid; FA: ferulic acid; *p*-CA: *p*-coumaric acid; CA: cinnamic acid; RT: rutin; QE: quercetin. ^3^ Means ± standard deviations followed by the same letters in the same column are not significantly different at the 0.05 probability level as determined by LSD. ^4^ n.d., not detected within the detection limit of the method.

**Table 8:** Pearson correlation coefficients between original variables and principal components, based on the combined data of lettuce grown under salt stress and treated with n-BC.

Variables	PC1 (54.29%)	PC2 (37.38%)	Variables	PC1 (54.29%)	PC2 (37.38%)
Plant height	0.64	0.72	Total soluble protein	−0.69	−0.17
Plant width	0.14	0.68	H_2_O_2_	0.20	−0.94
Number of leaves per plant	0.01	0.73	MDA	0.57	−0.64
Shoot fresh weight	0.55	0.72	APX	−0.96	0.04
Shoot dry weight	−0.59	0.56	CAT	−0.90	−0.07
Root fresh weight	−0.36	0.76	SOD	−0.95	−0.05
Root dry weight	−0.55	0.61	TPC	−0.92	0.09
Chla	−0.84	0.25	TFC	−0.92	0.13
Chlb	0.08	0.78	ABTS	−0.97	0.03
TChl	−0.68	0.59	DPPH	−0.89	0.25
Caro	0.20	0.90	CGA	−0.93	−0.18
Na^+^	−0.57	−0.79	CFA	−0.88	−0.35
K^+^	0.33	0.89	FA	−0.88	0.09
Na^+^/K^+^	−0.19	−0.97	*p*-CA	−0.95	−0.20
Ca^2+^	0.26	0.91	CA	−0.85	−0.07
Mg^2+^	0.06	0.96	RT	−0.84	−0.11
Proline	−0.88	0.30	QE	0.77	−0.43
Total soluble sugar	−0.91	0.23			

Correlation strength was classified as strong (>0.70), moderate (0.40–0.69), or weak (<0.40).

## Data Availability

The original contributions presented in this study are included in the article/Supplementary Material. Further inquiries can be directed to the corresponding author.

## Supplementary Materials

The following supporting information can be downloaded at: https://www.mdpi.com/article/10.3390/plants15010009/s1. Table S1: pH, EC, and element composition of biochar and nano-biochar derived from bamboo. Figure S1: Dynamic light scattering (DLS) results for n-BC.

## Abbreviations

The following abbreviations are used in this manuscript:

ABTS	2,2′-azino-bis(3-ethylbenzothiazoline-6-sulfonic acid)
ANOVA	Analysis of variance
APX	Ascorbate peroxidase
BC	Biochar
CA	Cinnamic acid
Caro	Carotenoid
CAT	Catalase
CFA	Caffeic acid
Chla	Chlorophyll a
Chlb	Chlorophyll b
CGA	Chlorogenic acid
CRD	Completely randomized design
DAT	Day after transplanting
DE	Dry extract
DLS	Dynamic light scattering
DPPH	2,2-diphenyl-1-picrylhydrazyl
DW	Dry weight
EC	Electrical conductivity
FA	Ferulic acid
FTIR	Fourier-transform infrared spectroscopy
GAE	Gallic acid equivalent
H_2_O_2_	Hydrogen peroxide
H_2_SO_4_	Sulfuric acid
HPLC	High-performance liquid chromatography
KI	Potassium iodide
LSD	Least significant difference
MDA	Malondialdehyde
n-BC	Nano-biochar
NPs	Nanoparticles
•O_2_^−^	Superoxide radical
PAL	Phenylalanine ammonia-lyase
PCA	Principal component analysis
*p*-CA	*p*-Coumaric acid
POD	Peroxidase
PVP	Polyvinylpyrrolidone
QE	Quercetin
QEq	Quercetin equivalent
ROS	Reactive oxygen species
RT	Rutin
SEM–EDS	Scanning electron microscopy with energy-dispersive X-ray spectroscopy
SiNPs	Silica nanoparticles
SOD	Superoxide dismutase
TBA	Tributylamine
TCA	Trichloroacetic acid
TChl	Total chlorophyll
TE	Trolox equivalent
TFC	Total flavonoid content
TPC	Total phenolic content

## References

[1] Kopecká R., Kameniarová M., Černý M., Brzobohatý B., Novák J. (2023). Abiotic stress in crop production. Int. J. Mol. Sci..

[2] Zhang L., Miras-Moreno B., Yildiztugay E., Ozfidan-Konakci C., Arikan B., Elbasan F., Ak G., Rouphael Y., Zengin G., Lucini L. (2021). Metabolomics and physiological insights into the ability of exogenously applied chlorogenic acid and hesperidin to modulate salt stress in lettuce distinctively. Molecules.

[3] Ahmad S., Khan S.A., Hussain A., Zhang L., Alomrani O.S., Ahmad A., Al-Ghanim K.A., Alshehri M.A., Ali S., Sarker P.K. (2024). Salt stress amelioration and nutrient strengthening in spinach (*Spinacia oleracea* L.) via biochar amendment and zinc fortification: Seed priming versus foliar application. Sci. Rep..

[4] Acosta-Motos J.R., Ortuño M.F., Bernal-Vicente A., Diaz-Vivancos P., Sanchez-Blanco M.J., Hernandez J.A. (2017). Plant responses to salt stress: Adaptive mechanisms. Agronomy.

[5] Munns R., Tester M. (2008). Mechanisms of Salinity Tolerance. Annu. Rev. Plant Biol..

[6] Isayenkov S.V., Maathuis F.J.M. (2019). Plant salinity stress: Many unanswered questions remain. Front. Plant Sci..

[7] van Zelm E., Zhang Y., Testerink C. (2020). Salt tolerance mechanisms of plants. Annu. Rev. Plant Biol..

[8] Shahzadi J., Zaib-un-Nisa A.N., Ali1 N., Iftikhar M., Shah A.A., Ashraf M.Y., Chao C., Shaffique S., Gatasheh M.K. (2025). Foliar application of nano biochar solution elevates tomato productivity by counteracting the effect of salt stress insights into morphological physiological and biochemical indices. Sci. Rep..

[9] Dhuldhaj U.P., Shaikh A.B., Puri J. (2022). Nano-biochar production and its characteristics: Overview. Int. J. Adv. Biol. Biomed. Res..

[10] Li P., Yan M., Li M., Zhou T., Li H., Si B. (2024). Migration rules and mechanisms of nano-biochar in soil columns under various transport conditions. Nanomaterials.

[11] Aziz S., Uzair B., Ali M.I., Anbreen S., Umber F., Khalid M., Alaa A.A., Mishra A.Y., Mishra V., Serrano-Aroca Á. (2023). Synthesis and characterization of nanobiochar from rice husk biochar for the removal of safranin and malachite green from water. Environ. Res..

[12] Ng L.Y.F., Ariffin H., Yasim-Anuar T.A.T., Farid M.A.A., Hassan M.A. (2022). High-energy ball milling for high productivity of nanobiochar from oil palm biomass. Nanomaterials.

[13] Yang H., Yang J., Liu L., Wang B. (2025). Nano-biochar derived from bamboo biomass: A dual-functional material for electrochemical sensing of ferulic acid and adsorptive removal of surfactants in cosmetic wastewater. Int. J. Electrochem. Sci..

[14] Zha Y., Zhao B., Niu T. (2022). Bamboo biochar and Zinc oxide nanoparticles improved the growth of maize (*Zea mays* L.) and decreased cadmium uptake in Cd-contaminated soil. Agriculture.

[15] Alfei S., Pandoli O.G. (2024). Bamboo-based biochar: A still too little-studied black gold and its current applications. J. Xenobiot..

[16] Xue N., Anwar S., Shafiq F., Gul-e-Kainat, Ullah K., Zulqarnain M., Haider I., Ashraf M. (2023). Nanobiochar application in combination with mulching improves metabolites and curd quality traits in cauliflower. Horticulturae.

[17] Khaliq H., Anwar S., Shafiq F., Ashraf M., Zhang L., Haider I., Khan S. (2023). Interactive effects of soil and foliar-applied nanobiochar on growth, metabolites, and nutrient composition in *Daucus carota*. Plant Growth Regul..

[18] Kumar A., Joseph S., Graber E.R., Taherymoosavi S., Mitchell D.R.G., Munroe P., Tsechansky L., Lerdahl O., Aker W., Sæbø M. (2021). Fertilizing behavior of extract of organomineral-activated biochar: Low-dose foliar application for promoting lettuce growth. Chem. Biol. Technol. Agric..

[19] Sultan H., Li Y., Ahmed W., Yixue M., Shah A., Faizan M., Ahmad A., Abbas H.M.M., Nie L., Khan M.N. (2024). Biochar and nano biochar: Enhancing salt resilience in plants and soil while mitigating greenhouse gas emissions: A comprehensive review. J. Environ. Manag..

[20] Mehr-un-Nisa, Shafiq F., Anwar S., Mahmood A., Iabal M., Ullah A., Zulqarnain M., Haider I., Ashraf M., Zhang L. (2023). Physiological effects of some engineered nanomaterials on radish (*Raphanus sativus* L.) intercropped with pea (*Pisum sativum* L.). Environ. Sci. Pollut. Res..

[21] Shani M.Y., Ahmad S., Ashraf M.Y., Nawaz M., Arshad I., Anjum A., De Mastro F., Cocozza C., Khan Z., Gul N. (2024). Nano-biochar suspension mediated alterations in growth, physio-biochemical activities and nutrient content in wheat (*Triticum aestivum* L.) at the vegetative stage. Plants.

[22] Rasheed A., Anwar S., Shafiq F., Zaib-un-Nisa, Khan S., Ashraf M. (2024). Physiological and biochemical effects of biochar nanoparticles on spinach exposed to salinity and drought stresses. Environ. Sci. Pollut. Res..

[23] Raza M.A.S., Shah A.N., Shahid M.A., Nawaz M., Ibrahim M.A., Iqbal R., Aslam M.U., Ercisli S., Ali Q. (2023). Nano-biochar enhances wheat crop productivity by vindicating the effects of drought: In relation to physiological and phenological stages. ACS Omega.

[24] Yousaf W., Shah A.A., Afzal M.B., Zaib-un-Nisa, Ali N., Ashraf M.Y., Elansary H.O., Ahmad A. (2024). Supplementation of nano-biochar improved growth and physiological attributes in wheat seedlings exposed to salt stress through enhanced activity of hydrolysing and nitrogen metabolic enzymes and regulation of crucial metabolites. S. Afr. J. Bot..

[25] Tourajzadeh O., Piri H., Naserin A., Cahri M.M. (2024). Effect of nano biochar addition and deficit irrigation on growth, physiology and water productivity of quinoa plants under salinity conditions. Environ. Exp. Bot..

[26] Gan P., Chen Y., Li Z., Li Y., Liang J., Zhao Z. (2025). Ferrihydrite-Biochar Augments Ecological Restoration in Reclaimed Coral Islands via Dual Pathways: Antioxidant System Stimulation and Microbial Metabolic Mediated C/N Cycling. Environ. Res..

[27] Simko I., Zhao R., Peng H. (2025). Differential impact of SiO_2_ foliar application on lettuce response to temperature, salinity, and drought stress. Plants.

[28] Santander C., Vidal G., Ruiz A., Vidal C., Cornejo P. (2022). Salinity eustress in seaweeds creases the biosynthesis and accumulation of phenolic compounds that improve the functional and antioxidant quality of red lettuce. Agronomy.

[29] Chaturvedi K., Singhwane A., Dhangar M., Milli M., Gorhae N., Niak N., Prashant N., Srivastava A.K., Verma M. (2024). Bamboo for producing charcoal and biochar for versatile applications. Biomass Convers. Biorefin..

[30] Raczkiewicz M., Ostolska I., Mašek O., Oleszczuk P. (2024). Effect of the pyrolysis conditions and type of feedstock on nanobiochars obtained as a result of ball milling. J. Clean. Prod..

[31] Freddo A., Cai C., Reid B.J. (2012). Environmental contextualisation of potential toxic elements and polycyclic aromatic hydrocarbons in biochar. Environ. Pollut..

[32] Lyu H., Gao B., He F., Zimmerman A.R., Ding C., Huang H., Tang J. (2018). Effects of ball milling on the physicochemical and sorptive properties of biochar: Experimental observations and governing mechanisms. Environ. Pollut..

[33] Pattnaik D., Kumar S., Bhuyan S.K., Mishra S.C. (2018). Effect of carbonization temperatures on biochar formation of bamboo leaves. IOP Conf. Ser. Mater. Sci. Eng..

[34] Francioso O., Schiavon M., Nardi S., Castellani D., Ferrari E., Estrada M.T.R., della Lucia M.C., Zuffi V., Ertani A. (2024). Mitigation of salt stress in *Lactuca sativa* L. var. Gentile Rossa using microalgae as priming agents. Plants.

[35] Dong X., Sun L., Guo J., Liu L., Han G., Wang B. (2021). Exogenous boron alleviates growth inhibition by NaCl stress by reducing Cl^−^ uptake in sugar beet (*Beta vulgaris*). Plant Soil.

[36] Zhou W., Zheng W., Wang W., Lv H., Liang B., Li J. (2022). Exogenous pig blood-derived protein hydrolysates as a promising method for alleviation of salt stress in tomato (*Solanum lycopersicum* L.). Sci. Hortic..

[37] Ramanayaka S., Kumar M., Etampawala T., Vithanage M. (2020). Macro, colloidal and nanobiochar for oxytetracycline removal in synthetic hydrolyzed human urine. Environ. Pollut..

[38] Hong J., Wang C., Wagner D.C., Gardea-Torresdey J.L., He F., Rico C.M. (2021). Foliar application of nanoparticles: Mechanisms of absorption, transfer, and multiple impacts. Environ. Sci. Nano.

[39] Wu M.Y. Effects of incorporation of nano-carbon into slow-released fertilizer on rice yield and nitrogen loss in surface water of paddy soil. Proceedings of the Third International Conference on Intelligent System Design and Engineering Applications.

[40] Djanaguiraman M., Anbazhagan V., Dhankher O.P., Prasad P.V.V. (2024). Uptake, translocation, toxicity, and impact of nanoparticles on plant physiological processes. Plants.

[41] Zhao Y., Yu H., Zhou J.M., Smith S.M., Li J. (2020). Malate circulation: Linking chloroplast metabolism to mitochondrial ROS. Trends Plant Sci..

[42] Taïbi K., Taïbi F., Abderrahim L.A., Ennajah A., Belkhodja M., Mulet J.M. (2016). Effect of salt stress on growth, chlorophyll content, lipid peroxidation and antioxidant defence systems in *Phaseolus vulgaris* L.. S. Afr. J. Bot..

[43] Murtaza G., Deng G., Usman M., Jamil A., Qasim M., Iqbal J., Ercisil S., Akrak M.I., Rizwan M., Elshikh M.S. (2024). Impact of *Acacia*-derived biochar to mitigate salinity stress in *Zea mays* L. by morpho-physiological and biochemical indices. Sci. Rep..

[44] Akter M., Oue H. (2018). Effect of saline irrigation on accumulation of Na^+^, K^+^, Ca^2+^, and Mg^2+^ ions in rice plants. Agriculture.

[45] Yang Y., Ye C., Zhao M., Li J., Zhang X., Yang Z., Yang Z., Algopishi U.B., Ahmed W. (2025). Nanoparticles in sustainable agriculture: Enhancing nutrient use efficiency and abiotic stress resilience under climate change. Plant Stress.

[46] Li R., Zhang R., Li Y., Liu C., Wang P. (2024). Foliar uptake and distribution of metallic oxide nanoparticles in maize (*Zea mays* L.) leaf. Environ. Sci. Technol..

[47] Fareed S., Haider A., Ramzan T., Ahmad M., Younis A., Zulfiqar U., Rehman H., Waraich E.A., Abbas A., Chaudhary T. (2024). Investigating the growth promotion potential of biochar on pea (*Pisum sativum)* plants under saline conditions. Sci. Rep..

[48] Kaur G., Sanwal S.K., Kumar A., Pundir R.K., Yadav M., Sehrawat N. (2024). Role of osmolytes dynamics in plant metabolism to cope with salinity induced osmotic stress. Discov. Agric..

[49] Huang M., Yin X., Chen J., Cao F. (2021). Biochar application mitigates the effect of heat stress on rice (*Oryza sativa* L.) by regulating the root-zone environment. Front. Plant Sci..

[50] Gill S., Ramzan M., Naz G., Danish S., Ansari M.J., Salmen S.H. (2024). Effect of silicon nanoparticle-based biochar on wheat growth, antioxidants and nutrients concentration under salinity stress. Sci. Rep..

[51] Mehmood H.M., Ashraf M.Y., Almas H.I., Zaib-un-Nisa, Ali N., Khaliq B., Ansari M.A., Singh R., Gul S. (2024). Synergistic effects of soil and foliar nano-biochar on growth, nitrogen metabolism and mineral uptake in wheat varieties. J. King Saud Univ. Sci..

[52] Alharbi K., Rashwan E., Mohamed H.H., Awadalla A., Omara A.E.D., Hafez E.M., Alshaal T. (2022). Application of silica nanoparticles in combination with two bacterial strains improves the growth, antioxidant capacity and production of barley irrigated with saline water in salt-affected soil. Plants.

[53] Al-Zahrani H.S., Alharby H.F., Hakeem K.R., Rehman R.U. (2021). Exogenous application of zinc to mitigate the salt stress in *Vigna radiata* (L.) Wilczek—Evaluation of physiological and biochemical processes. Plants.

[54] Wang R., Gao M., Ji S., Wang S., Meng Y., Zhou Z. (2016). Carbon allocation, osmotic adjustment, antioxidant capacity and growth in cotton under long-term soil drought during flowering and boll-forming period. Plant Physiol. Biochem..

[55] Borbély P., Iqbal N., Czékus Z., Tari I., Poór P. (2025). Exogenous sodium nitroprusside alleviates salt-induced changes in photosynthesis of greenhouse tomato plants by leaf age-dependent manner. J. Plant Growth Regul..

[56] Harakotr B., Trisiri T., Charoensup L., Thepsilvisut O., Rithichai P., Suwor P., Jirakiattikul Y. (2025). Effects of protein hydrolysate derived from hempseed by-products on growth, mineral contents, and quality of greenhouse grown red oak lettuce. Horticulturae.

[57] El-Nakhel C., Pannico A., Kyriacou M.C., Giordano M., De Pascale S., Rouphael Y. (2019). Macronutrient deprivation eustress elicits differential secondary metabolites in red and green-pigmented butterhead lettuce grown in a closed soilless system. J. Sci. Food Agric..

[58] Soviguidi D.R.J., Pan R., Liu Y., Rao L., Zhang W., Yang X. (2021). Chlorogenic acid metabolism: The evolution and roles in plant response to abiotic stress. Phyton Int. J. Exp. Bot..

[59] Gulzar N., Kamili A.N., Shah M.A. (2021). Silicon, the multifunctional element in reducing biotic and abiotic stress in plants. Int. Res. J. Plant Sci..

[60] Sepasi M., Iranbakhsh A., Saadatmand S., Ebadi M., Ardebili Z.O. (2024). Silicon nanoparticles (SiNPs) stimulated secondary metabolism and mitigated toxicity of salinity stress in basil (*Ocimum basilicum*) by modulating gene expression: A sustainable approach for crop protection. Environ. Sci. Pollut. Res..

[61] Lanqing L., Yue X., Keyan C., Jun Z., Min W., Wenqiang W., Zhifan Z., Fan L., Yadong D., Yinghao F. (2024). Adsorption characteristics of ball milling-modified Chinese medicine residue biochar toward quercetin. ACS Omega.

[62] Rouphael Y., Carillo P., Garcia-Perez P., Cardarelli M., Senizza B., Miras-Moreno B., Colla G., Lucini L. (2022). Plant biostimulants from seaweeds or vegetal proteins enhance the salinity tolerance in greenhouse lettuce by modulating plant metabolism in a distinctive manner. Sci. Hortic..

[63] Harakotr B., Srijunteuk S., Rithichai P., Tabunhan S. (2019). Effects of light-emitting diode light irradiance levels on yield, antioxidants and antioxidant capacities of indigenous vegetable microgreens. Sci. Technol. Asia.

[64] Lichtenthaler H.K., Wellburn A.R. (1983). Determinations of total carotenoids and chlorophylls a and b of leaf extracts in different solvents. Biochem. Soc. Trans..

[65] Bates L.S., Waldren R.P., Teare I.D. (1973). Rapid determination of free proline for water-stress studies. Plant Soil.

[66] DuBois M., Gilles K.A., Hamilton J.K., Rebers P.A., Smith F. (1956). Colorimetric method for determination of sugars and related substances. Anal. Chem..

[67] Bradford M.M. (1976). A rapid and sensitive method for the quantitation of microgram quantities of protein utilizing the principle of protein-dye binding. Anal. Biochem..

[68] Billah M., Sajib S.A., Roy N.C., Rashid M.M., Reza M.A., Hasan M.M., Talukder M.R. (2020). Effects of DBD air plasma treatment on the enhancement of black gram (*Vigna mungo* L.) seed germination and growth. Arch. Biochem. Biophys..

[69] da Silva L.J., Dias D.C.F.D.S., Sekita M.C., Finger F.L. (2018). Lipid peroxidation and antioxidant enzymes of *Jatropha curcas* L. seeds stored at different maturity stages. Acta Sci. Agron..

[70] Heshmati S., Dehaghi M.A., Farooq M., Wojtyla Ł., Maleki K., Heshmati S. (2021). Role of melatonin seed priming on antioxidant enzymes and biochemical responses of *Carthamus tinctorius* L. under drought stress conditions. Plant Stress.

[71] Önder S., Önder D.G., Tonguç M. (2020). Determination of hydrogen peroxide content and antioxidant enzyme activities in safflower (*Carthamus tinctorius* L.) seeds after accelerated aging test. J. Nat. Appl. Sci..

[72] Zhang S.Z., Hua B.Z., Zhang F. (2008). Induction of the activities of antioxidative enzymes and the levels of malondialdehyde in cucumber seedlings as a consequence of *Bemisia tabaci* (Hemiptera: Aleyrodidae) infestation. Arthropod-Plant Interact..

[73] Jirakiattikul Y., Ruangnoo S., Sangmukdee K., Chamchusri K., Rithichai P. (2024). Enhancement of plumbagin production through elicitation in in vitro-regenerated shoots of *Plumbago indica* L.. Plants.

[74] Folin O., Ciocalteu V. (1927). On tyrosine and tryptophane determinations in proteins. J. Biol. Chem..

[75] Kubola J., Siriamornpun S., Meeso N. (2011). Phytochemicals, vitamin C and sugar content of Thai wild fruits. Food Chem..

[76] Re R., Pellegrini N., Proteggente A., Pannala A., Yang M., Rice-Evans C. (1999). Antioxidant activity applying an improved ABTS radical cation decolorization assay. Free Radic. Biol. Med..

[77] Brand-Williams W., Cuvelier M.E., Berset C. (1995). Use of a free radical method to evaluate antioxidant activity. LWT Food Sci. Technol..

