# Supplementary material for: Foliar Application of Bamboo-Derived Nano-Biochar Enhances Morphological and Biochemical Responses of Lettuce (Lactuca sativa L.) Under Salt Stress
Source: Plants (Basel). 2025 Dec 19;15(1):9. doi: 10.3390/plants15010009 (PMC12787378; doi:10.3390/plants15010009)
Supplement: Supplementary file 1 [file plants-15-00009-s001.zip › plants-4000633-supplementary.pdf]

Table S1. pH, EC, and element compositions of biochar (BC) and nano biochar (n-BC) derived from bamboo.

	**BC**	**n-BC**
pH	8.59	6.81
EC (dS m^{-1})	0.610	0.595
Element composition (%wt)		
C	79.65	77.85
N	5.43	4.58
O	16.17	14.32
Mg	0.04	0.10
Al	0.01	0.04
Si	0.02	0.18
P	nd [1/]	0.05
K	0.35	0.78
Ca	0.01	0.03
Fe	0.05	0.05
Cu	0.12	0.17

[1/] n.d., not detected within the detection limit of the method.

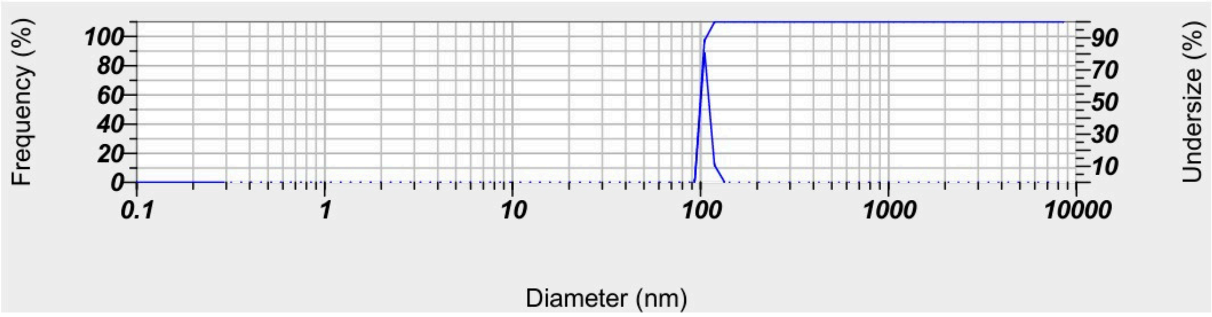

Figure S1: dynamic light scattering (DLS) results for n–BC.
